# Redefining the cerebral autoregulatory range of blood pressures: Not as wide as previously reported

**DOI:** 10.14814/phy2.15006

**Published:** 2021-08-26

**Authors:** Maria Jones‐Muhammad, Junie P. Warrington

**Affiliations:** ^1^ University of Mississippi Medical Center Program in Neuroscience Jackson MS USA; ^2^ Department of Neurology University of Mississippi Medical Center Jackson MS USA; ^3^ Department of Neurobiology & Anatomical Sciences University of Mississippi Medical Center Jackson MS USA

## Abstract

This editorial summarizes the manuscript by Brassard and colleagues entitled, “Losing the dogmatic view of cerebral autoregulation”. The main take‐home message is that the cerebral autoregulatory plateau is much smaller than previously accepted and needs to be re‐introduced as such.

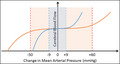

The brain is a highly metabolically active organ that utilizes ~20% of cardiac output at rest. While energy demand is high, overall brain perfusion remains relatively constant. This is thought to occur through modulating vessel diameter and thus, resistance. The classic cerebral autoregulation curve, described by Lassen (1959), showed that cerebral blood flow (CBF) is kept relatively constant at wide ranges of mean arterial pressures (MAP) of ~60–150 mmHg. Paulson later added the upper limit of the autoregulatory curve (Paulson et al., 1990) from animal studies, creating the autoregulatory curve that is widely used and cited in the literature (Figure 1, orange curve and shaded area). In the paper by Brassard et al. (2021) entitled, “*Losing the dogmatic view of cerebral autoregulation*”, the authors provide compelling evidence for updating the classic cerebral autoregulatory curve.

**FIGURE 1:**
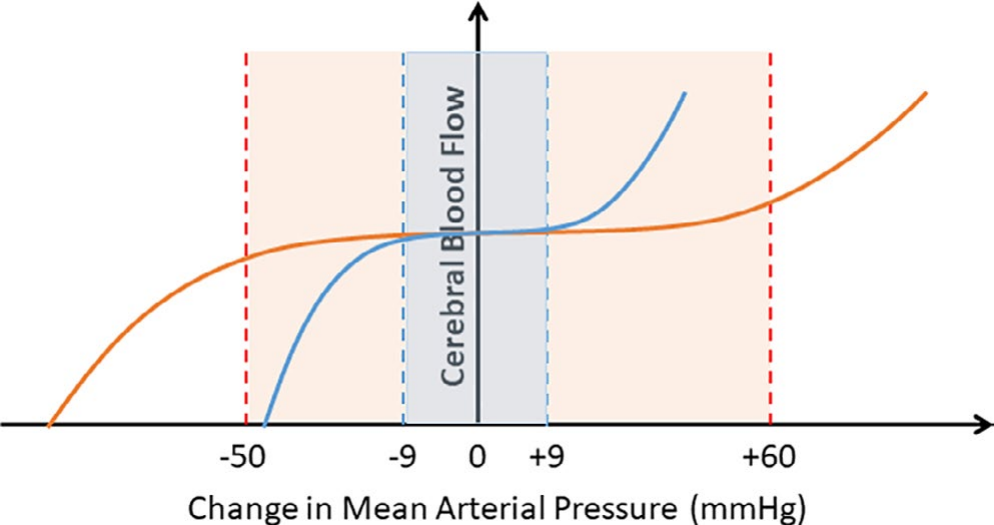
The cerebral autoregulatory range of blood pressure is much smaller than previously reported and accepted. The orange line and shaded area represents the cerebral autoregulatory curve and plateau from Lassen (1959) and Paulson et al. (1990). The blue line and shaded area represent the range of cerebral autoregulation reported from the reanalysis of the dataset from Numan et al. (2014), performed in the referenced manuscript by Brassard et al. (2021)

One of the arguments is that the autoregulatory curve was generated using unique data points (Lassen, 1959) from 376 subjects, obtained over seven different studies that included elevated blood pressures derived from administration of vasoactive drugs and patients with hypertensive disorders. While similar cerebral autoregulatory curves have been reproduced in different species such as rats and non‐human primates (Heistad et al., 1980; Hernandez et al., 1978), whether the ranges of cerebral autoregulation demonstrated by Lassen is applicable under non‐invasive, physiological circumstances has been disputed. Indeed, Heistad and Kontos (1983) challenged Lassen's study, highlighting that because some of the points on Lassen's curve were obtained using vasodilatory drugs known to have direct effects on CBF, one cannot claim that these specific points are representative solely of MAP‐dependent changes in CBF. Another concern was that three of the studies included in Lassen's curve showed increases in CBF with slight MAP changes, challenging whether there is a true plateau of CBF relative to MAP. Furthermore, the authors emphasize that optimally, cerebral perfusion pressure should be monitored in tandem with CBF, given that cerebral perfusion pressure changes with shifts in body position as well as pathologies. Thus, plotting changes in CBF relative to changes in cerebral perfusion pressure may be a better option.

Another key point raised by the authors is that previous work by Tan and colleagues showed that CBF is stable in healthy, non‐anesthetized individuals at a much lower range of MAP (~5–10 mmHg) than what was proposed by Lassen (~90 mmHg). To further extrapolate these findings, the authors performed a reanalysis of the dataset originally published by Numan et al. (2014) with the addition of recent papers published from 2012 to 2020. This resulted in a total of 29 papers with increased MAP and 27 papers with decreased MAP included in the analysis. Using a 3^rd^ order polynomial function to maximize goodness of fit to the curve, the reanalysis showed a smaller range of MAP for the CBF plateau. These data were analyzed with the exclusion of pharmacological intervention (Figure 1, blue curve and shaded area), thus removing the data points where vasoactive drugs could have contributed to confounding effects on CBF.

Brassard et al. (2021) also challenged whether a standard lower and upper range of cerebral autoregulation exists on an individual basis. The authors argue that an asymmetry in autoregulation, where the brain is better equipped to respond to increases in MAP but is not as efficient in adjusting to decreases in MAP, places the brain at greater risk of hypoperfusion and subsequent neurological deficits following drops in MAP (Selnes et al., 2012).

Instead of separating cerebral autoregulation into static (steady‐state) and dynamic (transient) processes, the authors suggest that the two processes should be viewed as a continuation of each other. They further elaborate that Lassen's curve reflects steady‐state, rather than physiological transient (dynamic) changes that tend to occur clinically. Thus, an important consideration is whether the cerebral autoregulatory curve, derived from observing slow changes in MAP, can and should be used in clinical settings where continuous blood pressure fluctuations are common.

Of course, limitations of available methodologies must also be considered. It is difficult to separate the direct effects of manipulations that directly or indirectly affect CBF (such as anesthesia) from the MAP‐induced effects. Additionally, there are limitations in the methodologies used to assess CBF. For example, transcranial Doppler ultrasound measures regional changes in blood velocities rather than global CBF changes (Willie et al., 2011). Moreover, magnetic resonance imaging lacks adequate temporal resolution and is therefore not useful for analyzing rapid changes in CBF (Kim et al., 1997).

In conclusion, this article challenges the current dogma of cerebral autoregulation based on Niels Lassen's work established over 60 years ago. The authors provide an insightful examination of several studies that challenge the use of the standard cerebral autoregulatory curve and provide a revised curve (Figure 1) based on almost a decade of findings, which indicate that CBF is constant at a lower range of MAP than previously thought and that autoregulation is more efficient when MAP is increased rather than decreased. This is highly relevant to clinical practice where the assumption of having a wide range of MAP where CBF remains constant can result in adverse clinical outcomes, especially where health professionals use the curve to find an “optimal” MAP at which to intervene (Zeiler et al., 2019). It is therefore imperative that cerebral autoregulatory range of blood pressure is redefined and that the upcoming generation of cerebrovascular scientists and physicians are (re)educated.

As with all challenges to accepted dogma, it may take some time for the concept of a smaller plateau of cerebral autoregulation to be accepted. Studies utilizing dynamic measures of CBF under physiological conditions (within subjects) and with transient changes in MAP will help solidify these findings. Nonetheless, we hope that the publication of this editorial along with the companion paper, will begin the conversation around this topic, and will spark further rigorous experiments in both humans and preclinical animal models.

## CONFLICT OF INTEREST

Authors have no conflicts to declare.

## AUTHOR CONTRIBUTIONS

Drafting and Writing: MJM; Revision: MJM and JPW; Approval: MJM and JPW.

## References

[phy215006-bib-0001] Brassard, P., Labrecque, L., Smirl, J. D., Tymko, M. M., Caldwell, H. G., Hoiland, R., Lucas, S. J. E., Denault, A. Y., Couture, E. J., & Ainslie, P. N. (2021). Losing the dogmatic view of cerebral autoregulation. Physiological Reports. 10.14814/phy2.14982 PMC8319534 34323023

[phy215006-bib-0002] Heistad, D., & Kontos, H. A. (1983). Cerebral Circulation, Supplement 8: Handbook of Physiology, The Cardiovascular System, Peripheral Circulation and Organ Blood Flow.

[phy215006-bib-0003] Heistad, D. D., Marcus, M. L., Piegors, D. J., & Armstrong, M. L. (1980). Regulation of cerebral blood flow in atherosclerotic monkeys. American Journal of Physiology, 239, H539–H544. 10.1152/ajpheart.1980.239.4.H539 6775542

[phy215006-bib-0004] Hernandez, M. J., Brennan, R. W., & Bowman, G. S. (1978). Cerebral blood flow autoregulation in the rat. Stroke, 9, 150–154. 10.1161/01.STR.9.2.150 644608

[phy215006-bib-0005] Kim, S. G., Richter, W., & Ugurbil, K. (1997). Limitations of temporal resolution in functional MRI. Magnetic Resonance in Medicine, 37, 631–636. 10.1002/mrm.1910370427 9094089

[phy215006-bib-0006] Lassen, N. A. (1959). Cerebral blood flow and oxygen consumption in man. Physiological Reviews, 39, 183–238. 10.1152/physrev.1959.39.2.183 13645234

[phy215006-bib-0007] Numan, T., Bain, A. R., Hoiland, R. L., Smirl, J. D., Lewis, N. C., & Ainslie, P. N. (2014). Static autoregulation in humans: a review and reanalysis. Medical Engineering & Physics, 36, 1487–1495. 25205587 10.1016/j.medengphy.2014.08.001

[phy215006-bib-0008] Paulson, O. B., Strandgaard, S., & Edvinsson, L. (1990). Cerebral autoregulation. Cerebrovascular and Brain Metabolism Reviews, 2, 161–192. 2201348

[phy215006-bib-0009] Selnes, O. A., Gottesman, R. F., Grega, M. A., Baumgartner, W. A., Zeger, S. L., & McKhann, G. M. (2012). Cognitive and neurologic outcomes after coronary‐artery bypass surgery. New England Journal of Medicine, 366, 250–257. 10.1056/NEJMra1100109 22256807

[phy215006-bib-0010] Willie, C. K., Colino, F. L., Bailey, D. M., Tzeng, Y. C., Binsted, G., Jones, L. W., Haykowsky, M. J., Bellapart, J., Ogoh, S., Smith, K. J., Smirl, J. D., Day, T. A., Lucas, S. J., Eller, L. K., & Ainslie, P. N. (2011). Utility of transcranial Doppler ultrasound for the integrative assessment of cerebrovascular function. Journal of Neuroscience Methods, 196, 221–237. 10.1016/j.jneumeth.2011.01.011 21276818

[phy215006-bib-0011] Zeiler, F. A., Ercole, A., Cabeleira, M., Carbonara, M., Stocchetti, N., Menon, D. K., Smielewski, P., & Czosnyka, M. (2019). Participants C‐THRS‐S, and Investigators . Comparison of performance of different optimal cerebral perfusion pressure parameters for outcome prediction in adult traumatic brain injury: A Collaborative European NeuroTrauma Effectiveness Research in Traumatic Brain Injury (CENTER‐TBI) Study. Journal of Neurotrauma, 36, 1505–1517. 30384809 10.1089/neu.2018.6182

